# *Ginkgo biloba* Extract Protects against Methotrexate-Induced Hepatotoxicity: A Computational and Pharmacological Approach

**DOI:** 10.3390/molecules25112540

**Published:** 2020-05-29

**Authors:** Lina Tariq Al Kury, Fazli Dayyan, Fawad Ali Shah, Zulkifal Malik, Atif Ali Khan Khalil, Abdullah Alattar, Reem Alshaman, Amjad Ali, Zahid Khan

**Affiliations:** 1 College of Natural and Health Sciences, Zayed University, Abu Dhabi 00000, UAE; Lina.AlKury@zu.ac.ae; 2 Riphah Institute of Pharmaceutical Sciences, Riphah International University, Islamabad 44000, Pakistan; dayyan.rph@gmail.com (F.D.); muhammadzulkifal95@gmail.com (Z.M.); 3 Department of Biological Sciences, National University of Medical Sciences, Rawalpindi 46000, Pakistan; Atif.ali@numspak.edu.pk; 4 Department of Pharmacology and Toxicology, Faculty of Pharmacy, University of Tabuk, 71491 Tabuk, Saudi Arabia; Aalattar@ut.edu.sa (A.A.); ralshaman@ut.edu.sa (R.A.); 5 Department of Botany, University of Malakand, Khyber Pakhtunkhwa 18800, Pakistan; amjad1990.aa48@gmail.co; 6 Department of Pharmacognosy, Faculty of Pharmacy, Federal Urdu University of Arts Science and Technology, Karachi 75300, Pakistan; Zahidkhan@fuuast.edu.pk

**Keywords:** *Ginkgo biloba*, hepatotoxicity, TNF-α, IL-1β, JNK, caspase-3, drug-protein interaction

## Abstract

*Ginkgo biloba* extract possess several promising biological activities; currently, it is clinically employed in the management of several diseases. This research work aimed to extrapolate the antioxidant and anti-inflammatory effects of *Ginkgo biloba* (Gb) in methotrexate (MTX)-induced liver toxicity model. These effects were analyzed using different in vivo experimental approaches and by bioinformatics analysis. Male SD rats were grouped as follows: saline; MTX; Gb (pretreated for seven days with 60, 120, and 180 mg/kg daily dose before MTX treatment); silymarin (followed by MTX treatment); Gb 180 mg/kg daily only; and silymarin only. Histopathological results revealed that MTX induced marked hepatic injury, associated with a substantial surge in various hepatic enzymes such as alanine transaminase (ALT), aspartate transaminase (AST), and serum alkaline phosphatase (ALP). Furthermore, MTX caused the triggering of oxidative distress associated with a depressed antioxidant system. All these injury markers contributed to a significant release of apoptotic (caspase-3 and c-Jun N-terminal kinases (JNK)) and tumor necrosis factor (TNF-α)-like inflammatory mediators. Treatment with Gb counteracts MTX-mediated apoptosis and inflammation dose-dependently along with modulating the innate antioxidative mechanisms such as glutathione (GSH) and glutathione S-transferase (GST). These results were further supplemented by in silico study to analyze drug-receptor interactions (for several Gb constituents and target proteins) stabilized by a low energy value and with a good number of hydrogen bonds. These findings demonstrated that Gb could ameliorate MTX-induced elevated liver reactive oxygen species (ROS) and inflammation, possibly by JNK and TNF-α modulation.

## 1. Introduction

The liver, as a critical part of the metabolic machinery, has a central role in homeostasis and detoxification of xenobiotics. Overproduction of intermediate toxic radicals, however, can disturb the innate antioxidant guard mechanism, leading to several pathological disorders of the liver such as acute and chronic hepatitis [1,2]. Furthermore, overwhelming levels of free radicals may cause the depletion of thiols and result in lipid peroxidation, leading to cell membrane damage and hepatic injury [3,4]. The inflammatory response develops secondary to tissue damage, which is triggered by these initial pathological events [5,6].

Methotrexate (MTX) is an anti-neoplastic chemotherapeutic drug, approved for several malignant conditions. MTX is also known to exhibit anti-inflammatory activity in conditions like psoriasis, rheumatoid arthritis, and Crohn’s disease, which further fortifies its usage [7]. However, high doses and prolonged use of MTX are associated with liver toxicity in humans, which is a major limitation of its use [8]. Consistently, the literature has suggested that the release of proinflammatory cytokines plays a critical role in the propagation of MTX-induced liver pathogenesis [9]. 

Natural products have been exploited as a repository for novel therapeutic identification for decades and thus have attracted considerable attention as a source of potential therapeutic agents. The antioxidant capabilities of many natural products have been consistently validated in experimental models. The hepatoprotective activities of various naturally derived compounds have been identified by several recent studies. For example, silymarin, extracted from *Silybum marianum*, or milk thistle, is a bioactive compound that has demonstrated predictable hepatoprotective effects in several animals and preclinical studies against a variety of insults, including MTX [10,11]. 

*Ginkgo biloba* (Gb) extract exhibits promising biological activities against neurodegenerative and vascular disorders [12,13]. The beneficial effects of Gb are due to its multi-component repository, in which flavonoids (25%), terpenoids (6%), and pro-anthocyanidins (7%) are the prominent components [14]. Furthermore, flavonoids have the potential to attenuate the majority of enzymes integrated into inflammatory cascades. Flavonoids also exert beneficial effects in cardiovascular diseases, possibly by inhibiting coagulation, thrombus formation, and platelet aggregation [15]. Terpenoids have been shown to suppress the nuclear factor-kB signaling in inflammation and cancer pathogenesis [16]. The beneficial hepatoprotective effects of Gb have been attributed to its modulating effect on endogenous antioxidant mechanisms, which were shown to critically regulate liver toxicity in several experimental models [17]. 

This research work aimed to investigate the hepatoprotective effects of *Ginkgo biloba* (Gb) in methotrexate (MTX)-induced liver toxicity model. We expect that the results of this study will help in identifying the cascading mechanisms involved in the hepatoprotective effect of Gb and thus provide a clue for multiple potential targeted therapeutics.

## 2. Material and Methods

All types of primary antibodies were purchased from Santa Cruz Biotechnology (SCBT, Santa Cruz, CA, USA). These include phosphorylated JNK (p-JNK), catalog number SC-6254; tumor necrosis factor (TNF-α), catalog number SC-52B83; cyclooxygenase-2 (COX-2), catalog number SC-514489; and caspase-3, catalog number SC-56053. Immunohistochemistry-related items such as Elite (Avidin/Biotin) system, catalog number SC-2018, and 3,3′-diaminobenzidine (DAB) reagent, catalog number SC-216567, were also obtained from Santa Cruz Biotechnology (SCBT, USA). A biotinylated goat anti-mouse was purchased from Abcam UK, with catalog number ab-6789. This antibody was used as a secondary antibody. Other chemicals like saline tablets, fixation solution (formaldehyde), antigen retrieval enzyme, quenching solvent (H_2_O_2_), and DPX mounting were ordered from BDH (Germany). Gb extract, methotrexate, glutathione (GSH), trichloroacetic acid (TCA), 1-chlor-2,4-dinitrobenzene (CDNP), N-(1-naphthyl)ethylenediamine dihydrochloride, 5,5′-dithio-bis-(2-nitrobenzoic acid) (DTNB), and silymarin were either a kind gift from local pharmaceutical industries, ensuring a highest analytical grade (Abbott and GSK Pharma, 99% HPLC grade), or purchased from Sigma.

### 2.1. Animals and Experimental Design

Sprague Dawley (SD) male rats weighing between 250 and 300 g and approximately 8–10 weeks old were acquired from an institutional breeding facility and were kept under a controlled environment at Riphah International University, Islamabad, Pakistan. The animals were maintained in plastic cages, under an equal light/dark period at room temperature with free access to food to facilitate experimental procedures. Extra care was practiced to avoid unnecessary stressful events. The investigational procedures were pre-endorsed from the Research and Ethics (REC) committee of Riphah International University, Islamabad, Pakistan, and as such strictly adhered to guidelines. Rats were divided into the saline, MTX, and Gb treatment groups (Gb was administered as 60, 120, or 180 mg/kg) and the silymarin group. Overall, a seven-day protocol was adopted, in which animals received either a single daily dose of saline (with 5% DMSO) or a daily oral dose of Gb (60, 120, or 180 mg/kg) or a daily dose of silymarin (100 mg/kg). MTX was administered on the 7th day as a single dose either after Gb administration or saline (disease group or MTX-only group).

**Figure 1:**
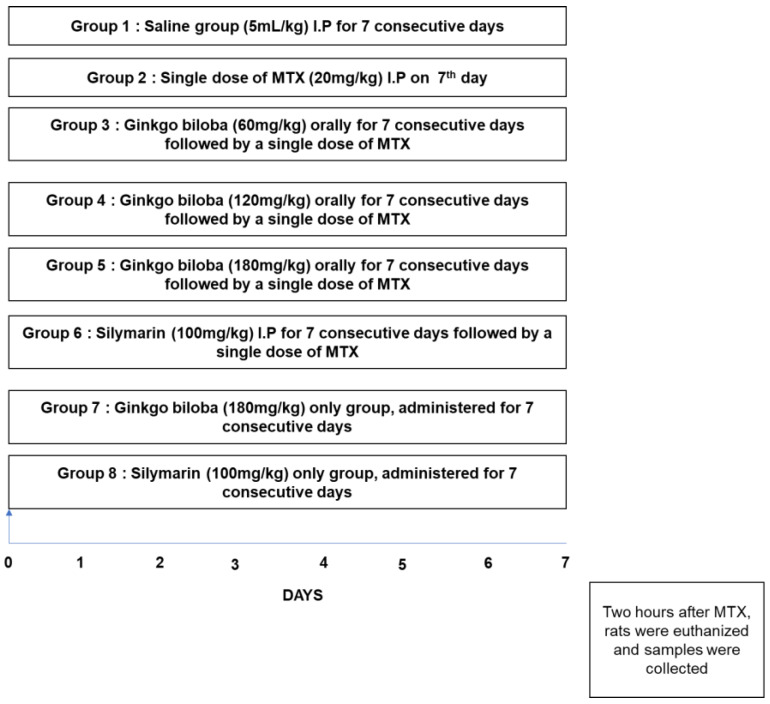
Diagrammatic design for the execution of the in vivo study. Rats were randomly grouped (n = 10 per group) as follows: (1) rats received a single daily dose of saline as a vehicle for 7 days; (2) rats were treated daily with saline and then on the 7th day received methotrexate (MTX) (20 mg/kg); (3) MTX + *Ginkgo biloba* (Gb) 60 mg, where rats received Gb (60 mg/kg) orally for 7 days and then on the 7th day received MTX (20 mg/kg); (4) MTX + Gb 120 mg, where rats were administered Gb (120 mg/kg) orally for 7 days and then on the 7th day received MTX (20 mg/kg); (5) MTX + Gb 180 mg, where rats received Gb (180 mg/kg) orally for 7 days and on the 7th day received MTX (20 mg/kg); (6) in the silymarin group, rats were administered silymarin (100 mg/kg) for 7 days and then and on the 7th day received MTX (20 mg/kg).

All drugs were dissolved in a mixture of 5% DMSO in saline. All animals that survived this period were utilized in the study. A total of four animals died during the experimental procedures, of which three were from the MTX-only group and one was from the low-dose Gb group; these groups were further adjusted by supplementing more animals. After 7 days (Figure 1), rats were anesthetized and divided into two cohorts (each cohort with n = 5 per group). One cohort was used for biochemical analysis: blood was taken from the heart, sera were obtained through centrifugation and preserved at –20 °C for subsequent analysis, and then liver tissues were analyzed for antioxidant assays. The biochemical determinants (alkaline phosphatase (ALP), total bilirubin, aspartate transaminase (AST), alanine transaminase (ALT)) were assessed by using standard commercial kits measured in the UV spectrophotometer. For the other cohort of animals, liver tissues from different groups were preserved in 4% paraformaldehyde for morphological analysis and later on processed for paraffin embedding and trimming by a microtome. Thin 4 μm sections were made and fixed on a coated slide.

### 2.2. Hematoxylin and Eosin (H&E) Staining

Tissue slides were subjected to deparaffinization protocol, which started with three different xylene treatments for 10 min followed by rehydration in graded alcohol preparation (commencing from 100% to 70%, each wash for several minutes). The slides were then rinsed with distilled water to clear any ethanol remaining and incubated for 10 min in hematoxylin at RT. After this, the slides were washed with distilled water and observed under the microscope to ensure nuclear staining. Otherwise, incubation time with hematoxylin was increased, followed by 1% HCl solution treatment for a short interval and then rinsing with distilled water, followed by immediate treatment with 1% ammonia water and then rinsing with water. The slides were then stained with pinkish eosin solution for the appropriate time and were then rinsed in water and kept under room temperature for air-drying. This step was followed by gradient ethanolic dehydration, fixation in absolute xylene (reverse deparaffinization protocol), and mounting with a glass coverslip. By using an Olympus light microscope at 40× magnification scale, slides were analyzed for the extent of neuronal death and survival using an ImageJ program [18].

### 2.3. Serum Biomarkers Analysis

The biochemical parameters or liver functional determinants (ALP, total bilirubin, AST, ALT) were determined by using standard commercial kits measured in the UV spectrophotometer. 

### 2.4. Oxidative Enzymes Analysis

Oxidative stress markers such as glutathione (GSH) level and glutathione transferase (GST) activity were determined to assess the degree of MTX damage and the relative effect of the test drug. After homogenization in phosphate-buffered saline (PBS) and centrifugation at 4000× *g* at 4 °C, the upper layer of clear supernatant was picked carefully. GSH level was assessed using a previously reported method with slight modifications. Here, 0.6 mM DTNB was dissolved in 0.2 M sodium phosphate, and then 2 mL of this solution was added to 0.2 mL of the supernatant. Then, 0.2 M PBS was added to make a final volume of 3 mL. The absorbance of the resultant mixture was measured after 10 min at 412 nm using a spectrophotometer. Phosphate buffer was used as blank whereas the DTNB solution was used as control. Real absorbance was calculated by subtracting the absorbance of control from that of the tissue lysate. Final GSH values were expressed in µmol/mg of proteins. For the determination of GST activity, three replicates of 1.2 mL reaction mixture were placed in a glass vial. Tissue supernatant was then added to the freshly prepared reaction mixture consisting of a 5:1 ratio of GST to CDNB. Blanks were also made using water by taking the same volume as the reaction mixture. Aliquots of 210 μL from the reaction mixture were pipetted in a 96-well plate, and the reaction rate was recorded using an ELISA reader at 340 nm. The GST activity was calculated using the extinction coefficient of the product formed and expressed as µmol of CDNB conjugate/min/mg of protein. For the nitric oxide assay, the previously reported protocols were adopted. Briefly, an equivalent quantity of tissue supernatant and saline was gently mixed with an equivalent quantity of Griess mixture, and the resulting mixture was further incubated. Absorbance was measured at 546 nm with an ELISA reader, using standard sodium nitrite solution to calibrate the absorbance coefficient.

### 2.5. Immunohistochemical Staining and Microscopic Analysis 

Immunohistochemical staining was achieved according to our previously published report [18]. Briefly, after the deparaffinization protocol described above, slides were treated with proteinase K to clear formaldehyde remains from the antigen epitope and then washed. A diluted H_2_O_2_ solution (3% in methanol) was employed for quenching peroxidase reactivity. After washing, slides were treated with a normal serum-like (NGS). Slides were then incubated for a whole night with primary antibodies, as demonstrated in material sections with a dilution factor of 1:100. The next morning, slides were consecutively treated with a biotin-tagged 2° antibody (dilution 1:50) and with an ABC kit (Santa Cruz Biotechnology) and then stained in DAB solution. This step was followed by gradient ethanolic dehydration and fixation in absolute xylene (reverse deparaffinization protocol) and mounting with a glass coverslip. By using an Olympus light microscope at 40× magnification scale, slides were analyzed for hyperactivated p-JNK, TNF-α, caspase-3, and COX2 using an ImageJ program [19].

### 2.6. Bioinformatics Resources

The bioinformatics study was done as described previously [20]. Briefly, 3D protein structures were modeled and passed through validation tools like Procheck [21] and ProSA [22]. By using AutoDock Vina, docking analysis was performed, for which Protein Data Bank (PDB) and mol2 files were generated for all model proteins and ligands, respectively. The docking results were interpreted by binding energies (E-value). From the E-value, the best ligand pose (orientation in protein after docking) was inferred using Discovery Studio Visualizer (DSV) in terms of ligand pose orientation and molecular interactions. 

### 2.7. Statistical Analysis

All data are represented as means ± standard error of the mean (SEM) and were analyzed by one-way ANOVA followed by Bonferroni multiple comparison post hoc testing via GraphPad Prism 7. ImageJ software was used for the analysis of morphological data.

## 3. Results

### 3.1. Effect of Gb on Liver Biochemical Markers

Biochemical detriments were assessed for the degree of MTX-induced hepatic toxicity (Table 1). A significant perturbation was demonstrated in the MTX-administered group, as evidenced by a marked increase in alanine aminotransferase (ALT) and aspartate aminotransferase (AST) (*p* < 0.001). A significant effect was also noticed for the bilirubin level (*p* < 0.05). Downregulation trends were observed for these biochemicals against different Gb doses (*p* < 0.05, Table 1).

### 3.2. Effect of Gb on Fatty Acid Levels

Generally, plasma levels of lipids and lipoproteins decrease with hepatic damage [23], which we likewise noticed in this study (Table 2). Furthermore, a downregulated trend for serum levels of triglycerides, cholesterol, and high-density lipoprotein (HDL) cholesterol was demonstrated as due to MTX. Treatment with Gb (60 and 120 mg) reversed the effect of MTX on triglycerides and low-density lipoprotein (LDL) cholesterol while causing no effect on HDL levels (Table 2).

### 3.3. Effect of Gb on Hepatic Oxidative Stress 

Table 3 shows the antioxidative enzyme changes following MTX administration. MTX treatment-induced ROS generation which is associated with the amassing of nitric oxide (NO) (100 ± 2.11 μmol/mg, *p* < 0.001) and the depletion of GST activity (8.6 ± 0.5 µmol CDNB conjugate/min/mg of protein, *p* < 0.001) and GSH level (30.3 ± 1.5 µmol/mg of protein, *p* < 0.001) relative to saline. All Gb doses used in this experiment attenuated MTX-induced downregulation of oxidative enzymes induced by MTX (*p* < 0.05).

### 3.4. Effect of Gb Liver Morphology 

**Figure 2:**
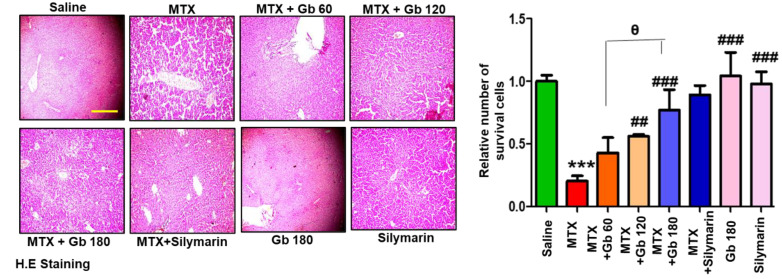
Gb restored the morphological integrity of the liver, as shown by histological examination. Liver tissue was stained with H&E (magnification, 20× scale bar 50 μm). Data are presented as means ± SEM and relative to saline. Data were analyzed by one-way ANOVA followed by Bonferroni multiple comparison post hoc testing using GraphPad Prism 5 software; *n* = 5 per group. The symbol ∗ shows a significant difference relative to saline, while # shows significant difference relative to the MTX group, while θ shows significant difference relative to treatment groups. Symbols ∗∗∗ or ### represent *p* < 0.001, while ## represents *p* < 0.01 values.

MTX administration caused detrimental disintegration of liver architecture (*p* < 0.001), an effect not evident in the saline group (Figure 2). The adverse effect of MTX could be traced near the area of the central vein, which is characterized by the inflammatory amassing of infiltrated cells and sinusoidal dilatation associated with hepatocyte degeneration. On the other hand, treatment with different doses of Gb restored the morphological alteration to a state appearing similar to the saline-treated group (Figure 2 and Table 4). 

### 3.5. Gb Attenuated MTX-Induced Liver Apoptosis

**Figure 3:**
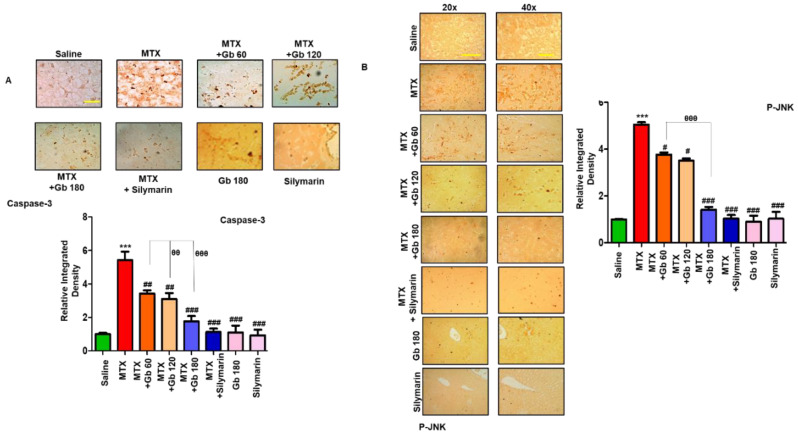
Gb attenuated apoptotic markers. The presented images indicate the immunoreactivity of (**A**) caspase-3 and (**B**) p-JNK. Scale bars = 50 μm (20× magnification) and 20 μm (40× magnification); n = 5 per group. Data presented are relative to saline, and the number of experiments performed was three. Data are presented as means ± SEM and were analyzed by one-way ANOVA followed by Bonferroni multiple comparison post hoc testing. The symbol ∗ shows a significant difference relative to saline, while # shows a significant difference relative to the MTX group. The symbols ∗∗∗ or ### or θθθ represent *p* < 0.001, while ## or θθ represent *p* < 0.01, while # shows *p* < 0.05.

JNK (p-JNK) is a triggering stimulus for the immunogenic response, including the effect on various proinflammatory cytokines [24]. Moreover, the role of JNK as an apoptotic marker is demonstrated in both extrinsic and intrinsic mitochondrial apoptotic pathways [25]. To investigate the anti-apoptotic role of Gb in our model, we performed immunostaining of p- JNK and caspase-3, as various degenerative models have shown the link between p-JNK and caspase activation, all linked to apoptotic cell death [26]. Compared to the saline group, we found a significant number of positive cells for p-JNK and caspase-3 (*p* < 0.001, Figure 3A,B). Gb treatment in a dose-dependent manner significantly reversed these apoptotic markers induced by MTX, as revealed by immunohistochemical analysis.

### 3.6. Gb Attenuated MTX-Induced Inflammatory Mediators in Liver

**Figure 4:**
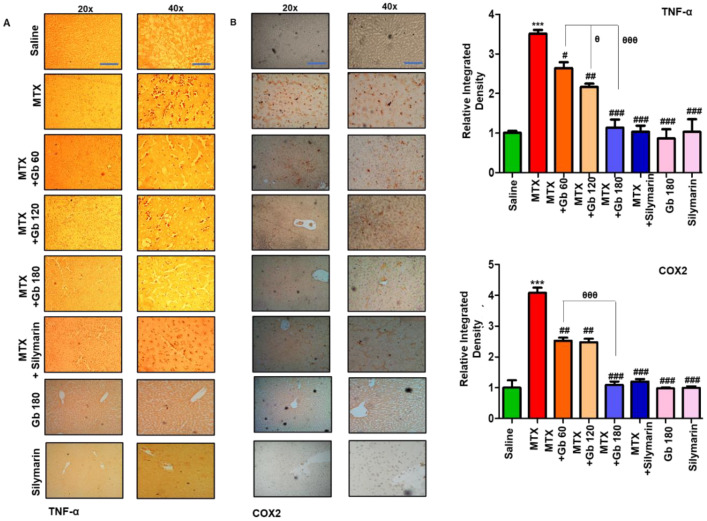
Gb attenuated inflammatory markers. The presented images indicate the immunoreactivity of (**A**) TNF-α and (**B**) COX2. Scale bars = 50 μm (20× magnification) and 20 μm (40× magnification); *n* = 5 per group. Data presented are relative to saline, and the number of experiments performed was three. Data are presented as means ± SEM and were analyzed by one-way ANOVA followed by Bonferroni multiple comparison post hoc testing. The symbol ∗ shows a significant difference relative to saline, while # shows a significant difference relative to the MTX group. Symbols ∗∗∗ or ### or θθθ represent *p* < 0.001, while ## represents *p* < 0.01, while # or θ show *p* < 0.05.

TNF-α is a proinflammatory cytokine that is immediately released from glia cell after neutrophil infiltration and thus plays a central role in mediating the inflammatory response [27]. Moreover, toll-like receptor 4 (TLR4) is located on glial cells, and its activation not only triggers TNF-α release but also leads to activation of several other potential downstream mediators, including iNOS, p-NFkB, and COX-2 [28]. We revealed whether MTX activates TLR4 and its downstream pathway by immunostaining of TNF-α and COX2. As expected, a higher expression was noticed in response to MTX (*p* < 0.001) (Figure 4A,B), whereas Gb treatment significantly reduced this hyperexpression (*p* < 0.01, Figure 4A,B). 

### 3.7. Docking Studies

**Figure 5:**
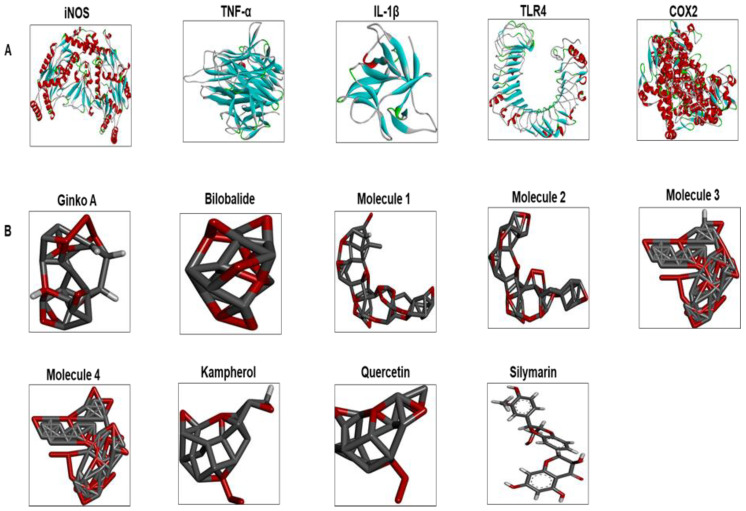
(**A**) The 3D structures of selected inflammatory protein targets. These are iNOS, TNF-α, IL-1β, TLR4, and COX2. (**B**) The different ligand structures (such as Gb constituents and silymarin) were made using ChemSketch and saved as a PDB file. iNOS: inducible nitric oxide; TLR4: toll-like receptor; COX2: cyclooxygenase; IL-1β: interleukin; TNF-α: tumor necrosis factor; Ginko A: ginkgolide A; Molecule 1: 3-O-(2′-O-(6′-O-(p-coumaroyl)-β-d-glucosyl)-α-l-rhamnosyl) kaempferol; Molecule 2: 3-O-(2′-O-(6′-O-(p-coumaroyl)-β-d-glucosyl)-α-l-rhamnosyl) quercetin; Molecule 3: 3-O-(2′-O,6′-O-bis (α-l-rhamnosyl)-β-d-glucosyl) kaempferol; Molecule 4: 3-O-(2′-O,6′-O-bis (α-l-rhamnosyl)-β-d-glucosyl) quercetin.

Ginkgolide A and bilobalide are chief constituents of terpene fractions which have previously demonstrated favorable biological activities [24,25]. Similarly, quercetin, kaempferol, and their glycosides are extensively studied in the literature. Based on previous reports and literature survey, we proceeded with these constituents for docking studies. The 3D structures of the modeled proteins, such as COX2, iNOS, TNF-ά, TLR4, IL-1β, and the drug constituents are shown in Figure 5.

**Figure 6:**
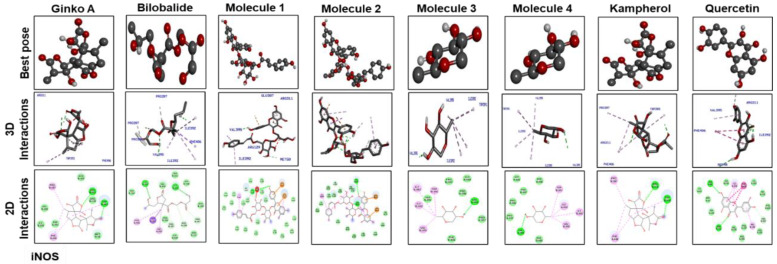
The best pose and docking results are presented here for ginkgolide A, bilobalide, 3-O-(2′-O-(6′-O-(p-coumaroyl)-β-d-glucosyl)-α-l-rhamnosyl) kaempferol (Molecule 1), 3-O-(2′-O-(6′-O-(p-coumaroyl)-β-d-glucosyl)-α-l-rhamnosyl)quercetin (Molecule 2), 3-O-(2′-O,6′-O-bis(α-l-rhamnosyl)-β-d-glucosyl) kaempferol (Molecule 3), 3-O-(2′-O,6′-O-bis(α-l-rhamnosyl)-β-d-glucosyl) quercetin (Molecule 4), quercetin, and kaempferol, which were docked into iNOS. Both 2D and 3D shapes of the drug-receptor complex were visualized by DSV.

The best pose and docking results are presented in Figure 6 for ginkgolide A, bilobalide, 3-O-(2′-O-(6′-O-(p-coumaroyl)-β-d-glucosyl)-α-l-rhamnosyl) kaempferol (Molecule 1), 3-O-(2′-O-(6′-O-(p-coumaroyl)-β-d-glucosyl)-α-l-rhamnosyl) quercetin (Molecule 2), 3-O-(2′-O,6′-O-bis (α-l-rhamnosyl)-β-d-glucosyl) kaempferol (Molecule 3), 3-O-(2′-O,6′-O-bis (α-L-rhamnosyl)-β-d-glucosyl) quercetin (Molecule 4), quercetin, and kaempferol. These constituents were docked against iNOS (Figure. 6). Table 4 shows the amino acid residues and binding energy values. Most of these ligands formed two hydrogen bonds with iNOS (Arg 311, Trp 393), except for Molecules 1, 3 and 4, which formed one hydrogen bond with Val 395. Furthermore, the hydrophobic interacting residues of iNOS are mostly the same for all the docking ligands.

**Figure 7:**
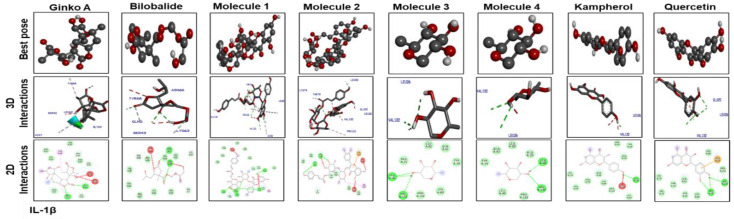
Docking results and best pose of ginkgolide A, bilobalide, 3-O-(2′-O-(6′-O-(p-coumaroyl)-β-d-glucosyl)-α-l-rhamnosyl)kaempferol (Molecule 1), 3-O-(2′-O-(6′-O-(p-coumaroyl)-β-d-glucosyl)-α-L-rhamnosyl) quercetin (Molecule 2), 3-O-(2′-O,6′-O-bis(α-l-rhamnosyl)-β-d-glucosyl) kaempferol (Molecule 3), 3-O-(2′-O,6′-O-bis(α-l-rhamnosyl)-β-d-glucosyl) quercetin (Molecule 4), quercetin, and kaempferol, after docking with IL-1β. Both 2D and 3D shapes of the drug-receptor complex were visualized by DSV.

**Figure 8:**
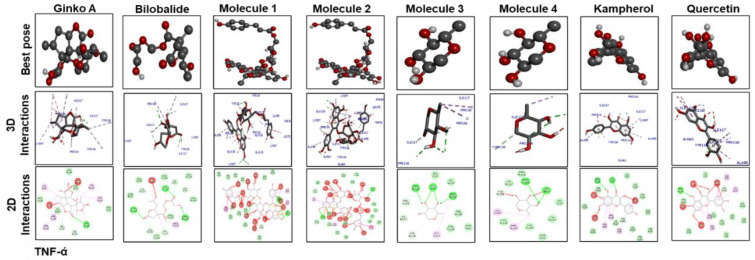
The different panels represent the docking analysis and best pose of ginkgolide A, bilobalide, 3-O-(2′-O-(6′-O-(p-coumaroyl)-β-d-glucosyl)-α-l-rhamnosyl) kaempferol (Molecule 1), 3-O-(2′-O-(6′-O-(p-coumaroyl)-β-d-glucosyl)-α-l-rhamnosyl) quercetin (Molecule 2), 3-O-(2′-O,6′-O-bis(α-l-rhamnosyl)-β-d-glucosyl)kaempferol (Molecule 3), 3-O-(2′-O,6′-O-bis(α-L-rhamnosyl)-β-d-glucosyl) quercetin (Molecule 4), quercetin, and kaempferol after docking with TNF-α. Both 2D and 3D shapes of the drug-receptor complex were visualized by DSV.

Figure 7 shows docking results with IL-1β. Ginkgolide A; bilobalide; Molecules 2, 3, and 4; and quercetin formed two hydrogen bonds with IL-1β. Leu 26 of IL-1β has ubiquitously formed hydrogen bonds with Molecules 1, 3, and 4; kaempferol; and quercetin. Moreover, Molecules 3 and 4, kaempferol, and quercetin have a similar binding pattern. Pro 116 and Lys 97 are the common binding residues of TNF-α involved in hydrogen bond formation with Molecules 3 and 4, kaempferol, quercetin, and bilobalide (Figure 8). Molecules 1 and 2 formed two hydrogen bonds with TNF-α (Glu115). Moreover, one hydrogen bond was formed between Tyr 118 of TNF-α and ginkgolide A. 

**Figure 9:**
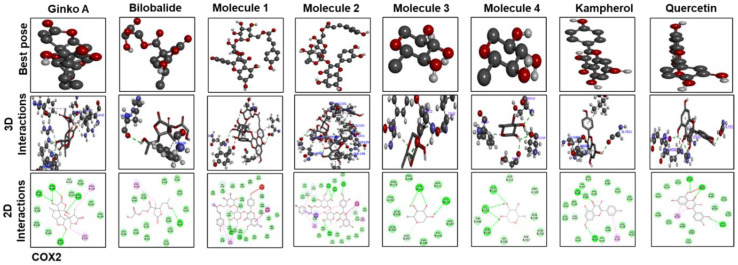
The different panels represent the docking analysis and best pose of ginkgolide A, bilobalide, 3-O-(2′-O-(6′-O-(p-coumaroyl)-β-d-glucosyl)-α-l-rhamnosyl) kaempferol (Molecule 1), 3-O-(2′-O-(6′-O-(p-coumaroyl)-β-d-glucosyl)-α-l-rhamnosyl) quercetin (Molecule 2), 3-O-(2′-O,6′-O-bis(α-L-rhamnosyl)-β-d-glucosyl) kaempferol (Molecule 3), 3-O-(2′-O,6′-O-bis(α-l-rhamnosyl)-β-d-glucosyl) quercetin (Molecule 4), quercetin, and kaempferol, after docking with COX2. Both 2D and 3D shapes of the drug-receptor complex were visualized by DSV.

**Figure 10:**
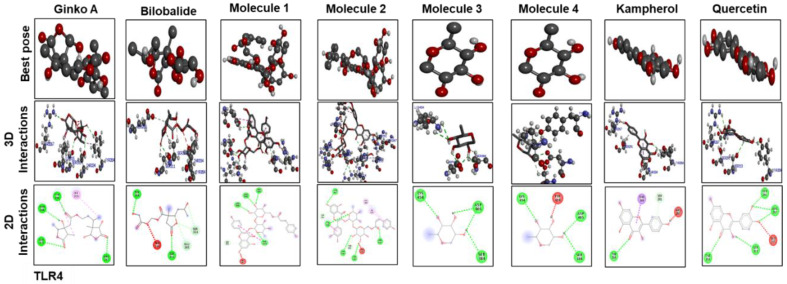
The different panels represent the docking analysis and best pose of ginkgolide A, bilobalide, 3-O-(2′-O-(6′-O-(p-coumaroyl)-β-d-glucosyl)-α-l-rhamnosyl) kaempferol (Molecule 1), 3-O-(2′-O-(6′-O-(p-coumaroyl)-β-d-glucosyl)-α-l-rhamnosyl) quercetin (Molecule 2), 3-O-(2′-O,6′-O-bis(α-l-rhamnosyl)-β-d-glucosyl) kaempferol (Molecule 3), 3-O-(2′-O,6′-O-bis(α-l-rhamnosyl)-β-d-glucosyl) quercetin (Molecule 4), quercetin, and kaempferol, after docking with TLR4. Both 2D and 3D shapes of the drug-receptor complex were visualized by DSV.

**Figure 11:**
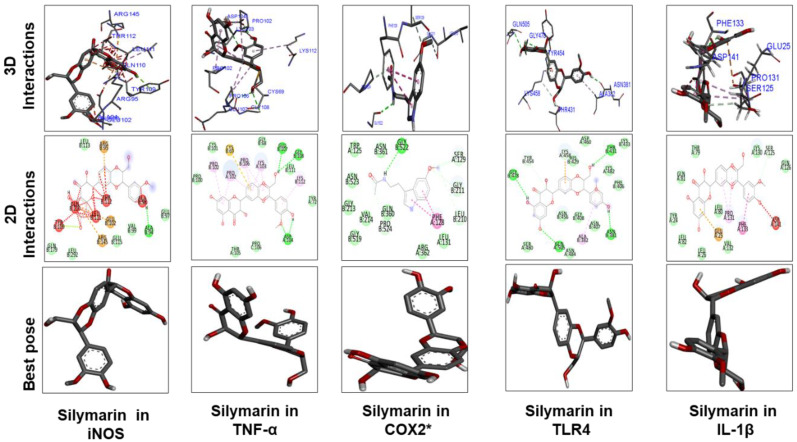
The different panels represent the docking analysis and best pose of silymarin that fitted to iNOS, TNFα, COX2, TLR4, and IL-1β. Both 2D and 3D shapes of the drug–receptor complex were visualized by DSV.

Figure 9 and Figure 10 show docking results of ginkgolide A; bilobalide; Molecules 1, 2, 3, and 4; kaempferol; and quercetin with COX2 and TLR4, respectively. The docking results demonstrated that Gb constituents are tightly connected to COX2 and TLR4. Furthermore, ginkgolide A linked with COX2 by 5 H-bonds and with TLR4 with 4 H-bonds. Quercetin linked to COX2 by 3 H-bonds and TLR4 by 5 H-bonds. Molecules 2 and 3 formed 5 and 4 hydrogen bonds, respectively, with TLR4. Figure 11 represents the docking results of silymarin with iNOS, TNF-α, COX2, TLR4, and IL-1β. Binding energy and amino acid residues involved in H-bond formation between different ligands and IL1-β, TNFα, COX2, and TLR4 are shown in Table 5.

Table 5Binding energy and amino acid residues involved in polar contacts between different ligands and IL1-β, TNFα, COX2, and TLR4.

**Table:** (**A**)

iNOS				IL1-β			TNFα		
Ligand	Binding Energy	# of H Bonds	Residue	Binding Energy	# of H Bonds	Residue	Binding Energy	# of H Bonds	Residue
Ginkgolide	–10	2	TRP393, ARG311	–8.9	2	GLY64, HIS7	–7.8	1	TYR118
Bilobalide	–9.8	2	ILE392,ILE392	–9.1	2	GLN5, SER43	–7.9	3	LYS97, PRO116, TYR118
Molecule 1	–9.1	1	VAL395	–6.3	6	LEU26, VAL132, THR79, LEU(80)2, LEU134	–7.4	3	TYR118, GLU(115)2
Molecule 2	–8.7	3	TRP393, ARG311(2)	–6.4	2	THR79, LEU82	–7	3	GLU115, GLN(61)2
Molecule 3	–8.8	1	VAL395	–6.2	3	LEU(20)2, VAL132	–6.3	4	PRO126, LYS(97)2, PRO116
Molecule 4	–7	1	VAL395	–6.6	2	LEU20, VAL132	–5.2	2	PRO116, LYS97
Kaempferol	–7.4	2	ARG311, TRP393	–6.5	1	LEU26	–7.2	2	LYS97, PRO116
Quercetin	–7.8	2	ARG311, SER48	–6.8	3	VAL132, LEU(26)2	–8.9	1	PRO116
Silymarin	–9.6	1	ALA94	–8	1	ASP141	–9.1	4	GLY108, GLU 107, ASP(164)2

**Table:** (**B**)

COX2				TLR4		
Ligand	Binding Energy	# of H Bonds	Residue	Binding Energy	# of H Bonds	Residue
Ginkgolide	–6.9	4	ASN(361)2, ARG(3622)	–7.9	4	ARG267, SER313, THR334, TYR354
Bilobalide	–7.3	1	HIS212	–8.1	2	TYR354, SER311
Molecule 1	–6.7	2	LEU210, HIS212	–7.3	4	TYR354, ASP350, ARG359, THR336
Molecule 2	–8.9	5	ARG362, ASN(361)2, SER129, GLN360	–8.4	5	ARG267, TYR354, ASP356, SER358, ARG316
Molecule 3	–6.3	3	SER(112)2, ILE110	–7.2	4	LYS454, ASP(405)2, SER 384
Molecule 4	–7.8	4	SER 112, ILE(110)2, SER 107	–6.6	3	ASP465, SER 384, LYS454
Kaempferol	–6.8	1	GLN 360	–6.1	1	TYR354
Quercetin	–6.4	3	GLY 211, ARG362, ASN 361	–7.8	4	TYR354, ARG(267)2, SER291
Silymarin	–5.8	1	GLY522	–8	4	THR381, GLN505, GLY478, ASN 381

## 4. Discussion

MTX is an anticancer drug, immunosuppressant, and disease-modifying anti-rheumatic agent, clinically indicated as useful for multiple human ailments [29]. However, consistent reports established liver toxicity as a major side effect of MTX administration. The proposed mechanism behind this pathogenesis is thought to be the enhancement of oxidative stress, although polyglutamate accumulation with subsequent folate depletion is another potential mechanism [30]. 

In agreement with earlier studies, we observed high serum levels of ALP, AST, ALT, and serum bilirubin in the MTX-intoxicated group [7]. Similarly, Gb administration reverted the toxic serum level of MTX, as low levels of AST and ALT were noted (Table 1), in line with previously reported data [17]. Furthermore, the liver is central to lipid and lipoprotein metabolism; therefore, previous reports demonstrated an altered level of lipids and their biometabolic products in liver damage [23]. Our work showed that MTX upregulated cholesterol, low-density lipoprotein cholesterol, and triglyceride (TG) (Table 2), which lead to exaggerated inflammatory reactions. Previous reports demonstrated that critical genes were expressed along with MTX usage, genes that modulate the synthesis of certain fatty acids, and are implicated in both liver and cardiovascular pathogenicity [31]. Some of these effects were attenuated in Gb pretreated groups; however, no effects were noticed on TG and HDL levels, which is in line with previously reported data [30].

GSH and GST are innate antioxidant enzymes that can overcome free radical formation (such as ROS and nitrates). It has been reported that MTX initiates oxidative distress both by augmenting free radical content and by diminishing hepatic antioxidant enzymes (Table 3) [32,33]. Furthermore, distinct features of hepatic injury, such as infiltration of neutrophils, congestion, apoptosis, and necrotic cell death, were evident in the MTX group, consistent with delineated biochemical changes (Figure 2). These pathophysiological alterations in serum and at tissue level were mitigated by Gb pretreatment, supporting its effective role in counteracting MTX-induced liver toxicity (Figure 2).

JNKs play a crucial role in oxidative-stress-induced apoptotic signaling, which can be implicated either by extrinsic or intrinsic pathways [34,35]. Furthermore, the role that MTX plays by inducing apoptosis through the mitochondrial extrinsic apoptotic pathway has also been reported [36,37]. Previous reports showed that the amplified expression of pro-apoptotic genes such as TNF-α, caspase-3, and COX-2 occurs due to the JNK pathway activation [36]. In the present study, we demonstrated higher p-JNK expression in the MTX group. Consistent with an earlier study, expression levels of JNK were significantly reduced by Gb pretreatment (Figure 3B) [38]. Our results demonstrated that MTX induced the release of TNF-α and COX-2, which was attenuated by Gb pretreatment, a consistent finding with previous reports showing that Gb extract possesses anti-inflammatory activity [39]. In support of our findings, Gb extract was found to ameliorates colitis and cause a release of proinflammatory mediators by LPS in mice [40]. All the above-mentioned promising beneficial effects indicate that Gb could impede several cascading pathways in this model of hepatotoxicity.

Docking analysis was performed to further demonstrate drug-protein interaction. No 3D structures are available for several rat proteins, including iNOS, TNF-α, IL-1β, and TLR4. Therefore, we have previously built the 3D structures of these proteins by homology modeling, further assessed for stability by molecular dynamics (MD) simulation. The modeled proteins were then subjected to docking analysis, where binding energy was evaluated and interactions were visualized in the Discovery Studio (DS). Gb bound each target protein by forming H-bonds and other hydrophobic interactions. Moreover, the role of hydrogen bonding in the drug-receptor complex is integral for complex stability, and our findings are consistent with previous literature [41].

In conclusion, this study demonstrated that Gb has a protective role in MTX-induced inflammation and apoptosis by reducing the expression levels of TNF-α, p-JNK, caspase-3, and COX-2 pathways in rat liver. By inhibiting ROS generation, elevating liver GSH and GST, and reducing NO level, Gb reduced hepatic oxidative damage dose-dependently. Further studies are required to fully understand the molecular mechanisms of the protective effects that Gb has in liver toxicity.

## Figures and Tables

**Table 1:** Effect of Gb on liver biochemical markers.

Treatment	ALT	AST	TOTAL BILIRUBIN	ALKALINE PHOSPHATASE
Saline (5 mL/kg)	22.0 ± 10	26.20 ± 0.58	0.78 ± 0.05	149 ± 18.09
MTX (20 mg/kg)	164.80 ± 4.25 ***	272.2 ± 13.22 ***	0.92 ± 0.06 *	158.20 ± 6.23
MTX + Gb (60 mg/kg)	37.20 ± 1.65 ^###^	132.40 ± 1.7 ^###^	0.70 ± 0.03	127.40 ± 6.61
MTX + Gb (120 mg/kg)	27.80 ± 1.65 ^###^	119.80 ± 1.56 ^###^	0.82 ± 0.06	136.40 ± 9.41
MTX + Gb (180 mg/kg)	35.20 ± 6.26 ^###^	151.80 ± 12.66 ^###^	0.80 ± 0.03	166.20 ± 7.21
MTX + Silymarin (100 mg/kg)	67.80 ± 6.74	37.00 ± 9.77	0.74 ± 0.05	142.80 ± 11.93

Data are shown as means ± standard error of the mean (SEM) and were analyzed by one-way ANOVA followed by Bonferroni multiple comparison post hoc testing using GraphPad Prism 7 software. Symbols *** or ### indicate *p* < 0.001. The symbol * shows a significant difference to saline, while # shows a significant difference to the MTX group; *n* = 5. AST: aspartate aminotransferase; ALT: alanine aminotransferase; MTX: methotrexate; Gb: *Ginkgo biloba*.

**Table 2:** Effect of Gb on fatty acid levels.

Treatment	Triglycerides	Cholesterol	LDL Cholesterol	HDL Cholesterol
Saline (5 mL/kg)	86.40 ± 2.69	95.40 ± 6.70	31.20 ± 0.66	18.00 ± 1.14
MTX (20 mg/kg)	67.60 ± 1.28*	70.00 ± 5.75*	43.00 ± 4.46*	14.40 ± 1.28*
MTX + Gb (60 mg/kg)	117.40 ± 3.23^#^	56.40 ± 2.92	19.20 ± 2.72^##^	14.20 ± 1.06
MTX + Gb (120 mg/kg)	102.80 ± 8.49^#^	55.80 ± 2.28	24.40 ± 2.24^##^	11.20 ± 0.58
MTX + Gb (180 mg/kg)	70.20 ± 5.77	63.80 ± 3.51	37.00 ± 3.42	13.60 ± 0.70
MTX + Silymarin (100 mg/kg)	88.20 ± 5.67	74.80 ± 2.08	37.80 ± 3.30	15.00 ± 0.31

Data are shown as means ± standard error of the mean (SEM) and were analyzed by one-way ANOVA followed by Bonferroni multiple comparison post hoc testing using GraphPad Prism 7 software. Symbols * or # represent *p* < 0.05, while ## represents *p* < 0.01. The symbol * shows a significant difference to saline, while # shows a significant difference to the MTX group; *n* = 5. LDL: Low-density lipoprotein; HDL: high-density lipoprotein; MTX: methotrexate; Gb: *Ginkgo biloba*.

**Table 3:** Effect of Gb on hepatic oxidative stress.

Treatment	GST µmol CDNB Conjugate/min/mg of Protein	NO μmol/mg	GSH µmol/mg of Protein
Saline (5 mL/kg)	55 ± 0.7	17 ± 2.5	85 ± 1.6
MTX (20 mg/kg)	8.6 ± 0.5***	100 ± 2.11***	30.3 ± 1.5***
MTX + Gb (60 mg/kg)	19.94 ± 0.13^#^	92.18 ± 2.15	68 ± 0.5^#^
MTX + Gb (120 mg/kg)	25.98 ± 0.3^#^	78.28 ± 5.7^##^	142 ± 6.8^###^
MTX + Gb (180 mg/kg)	31.24 ± 0.6^#^	58.30 ± 0.90^###^	161.5 ± 1^###^
MTX + Silymarin (100 mg/kg)	77.8 ± 0.5	20.567 ± 0.8	90.23 ± 0.3

Data are shown as means ± standard error of the mean (SEM) and were analyzed by one-way ANOVA followed by Bonferroni multiple comparison post hoc testing using GraphPad Prism 7 software. Symbols ∗∗∗ or ### represent *p* < 0.001, ## represents *p* < 0.01, and # represents *p* < 0.05. The symbol ∗ shows a significant difference to saline, while # shows a significant difference to the MTX group; *n* = 5 per group. GST: glutathione S-transferases; GSH: glutathione; NO: nitric oxide; MTX: methotrexate; Gb: *Ginkgo biloba*.

**Table 4:** Extent of liver damage by MTX.

Histopathology	Saline	MTX	MTX+ Gb 60	MTX+ Gb 120	MTX+ Gb 180	MTX+ Silymarin	Gb 180	Silymarin
Hepatoportal and Sinusoidal Cogestion	**−**	**++**	**+**	**+/−**	**−**	**−**	**−**	**−**
Apoptosis	**−**	**+++**	**++**	**+**	**+**	**−**	**−**	**−**
Necrotic damage	**−**	**++**	**+**	**−**	**−**	**−**	**−**	**−**
Inflammatory Infiltrate	**−**	**++**	**+**	**−**	**−**	**+/−**	**−**	**−**

Note: +++, maximum detrimental change; ++, moderate detrimental change; +, minimum detrimental change; +/−, less or no change; −, no change.
